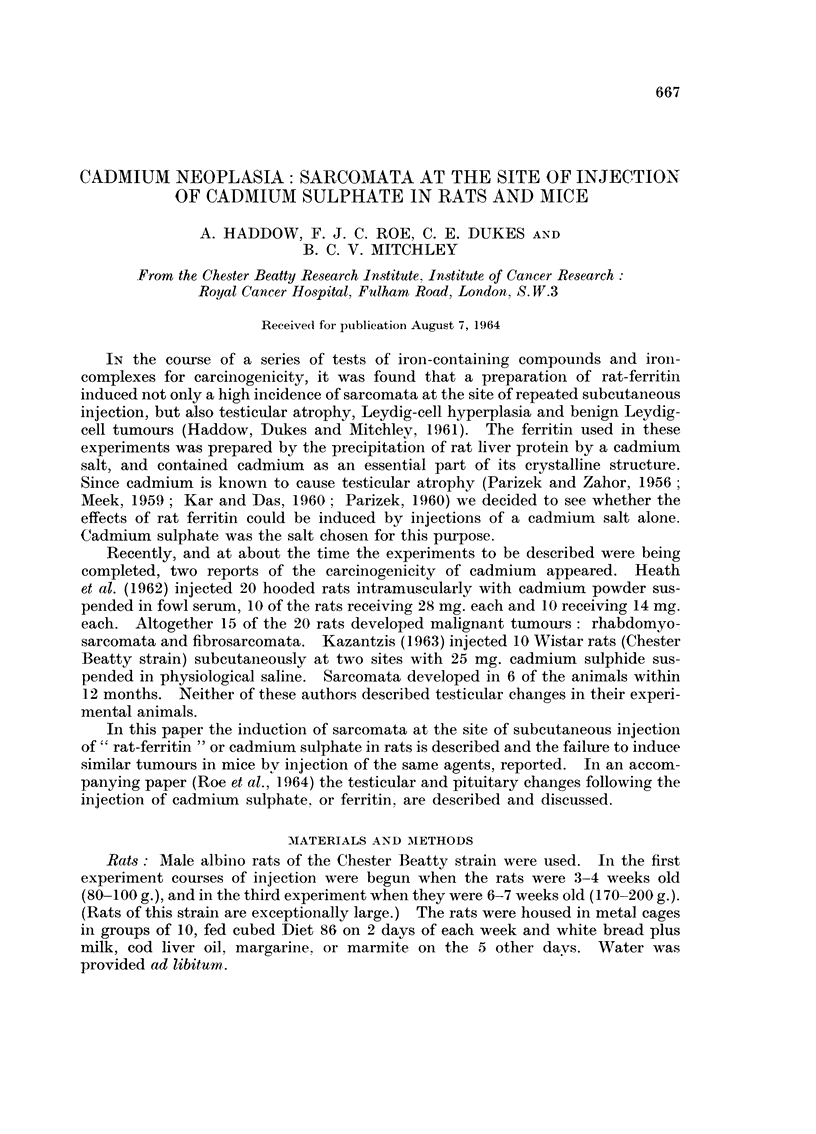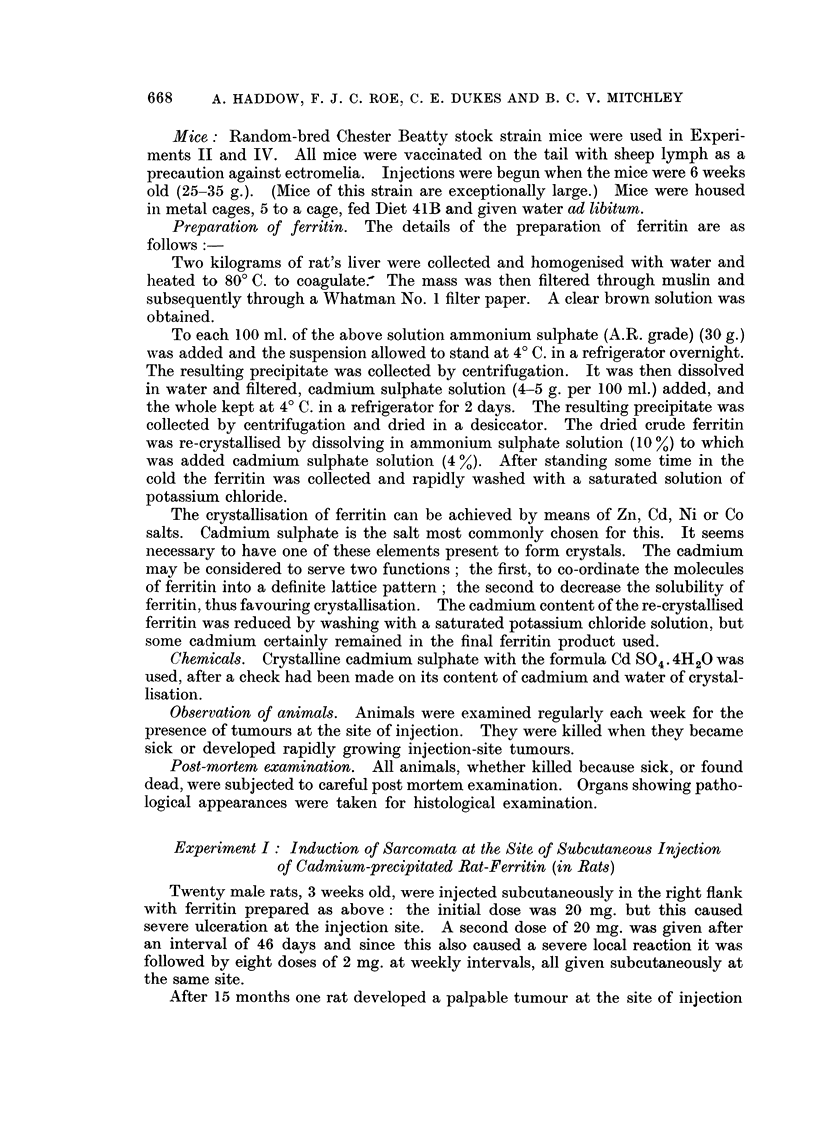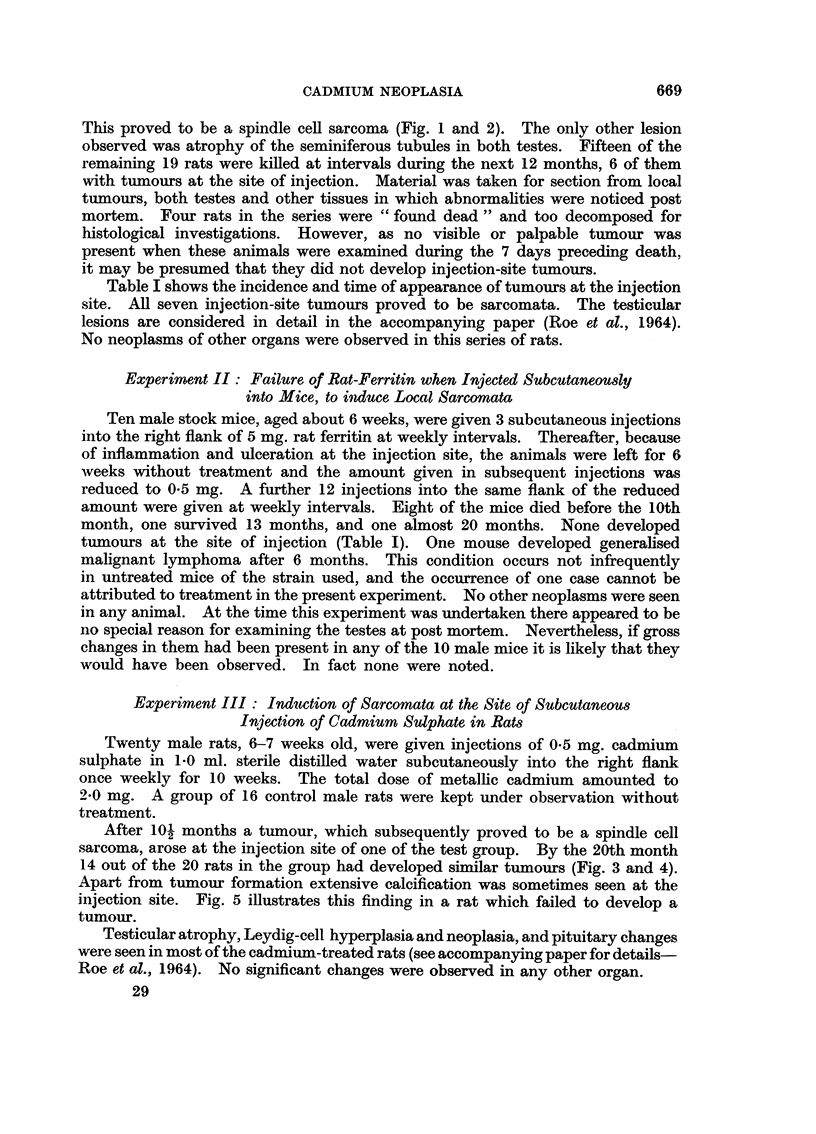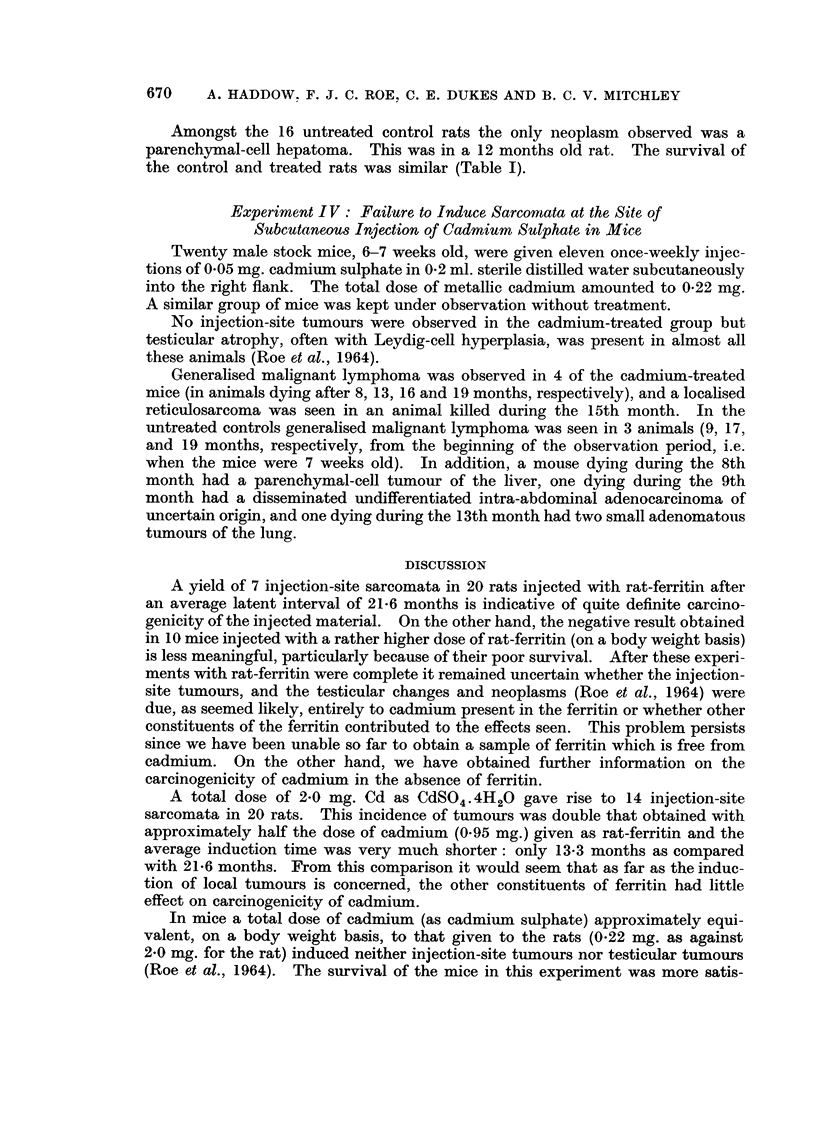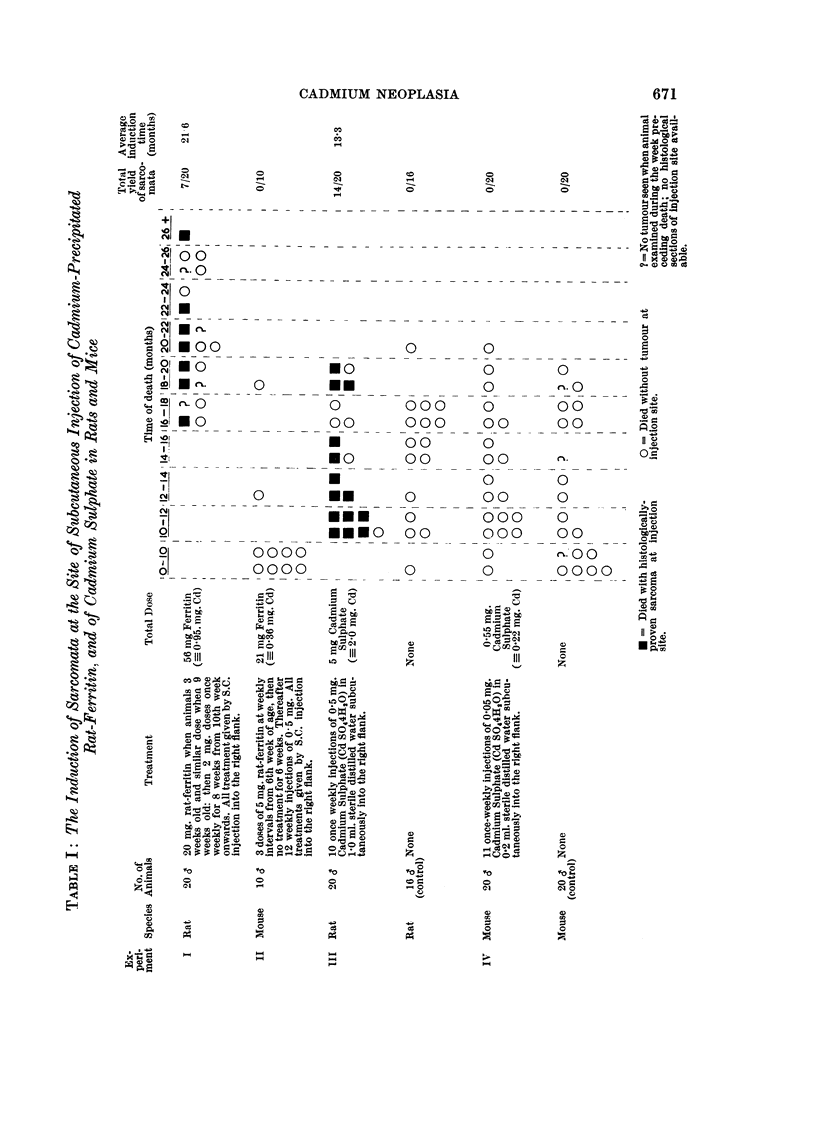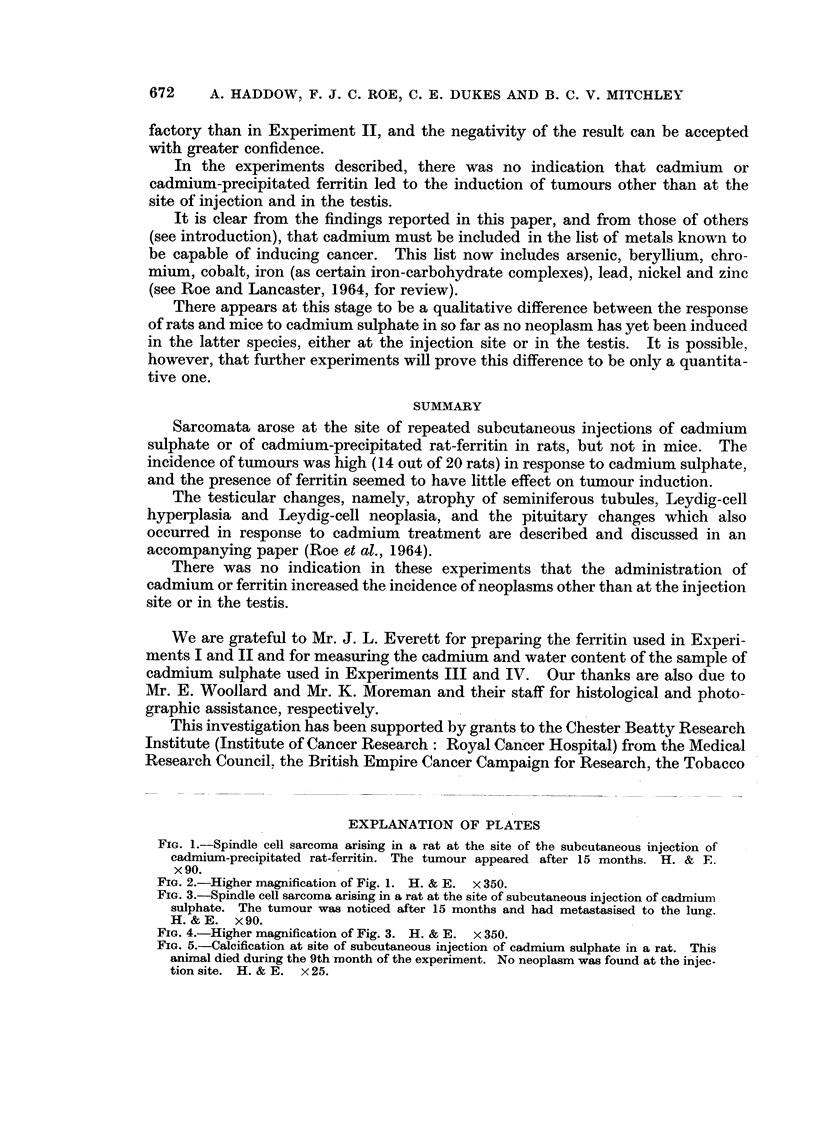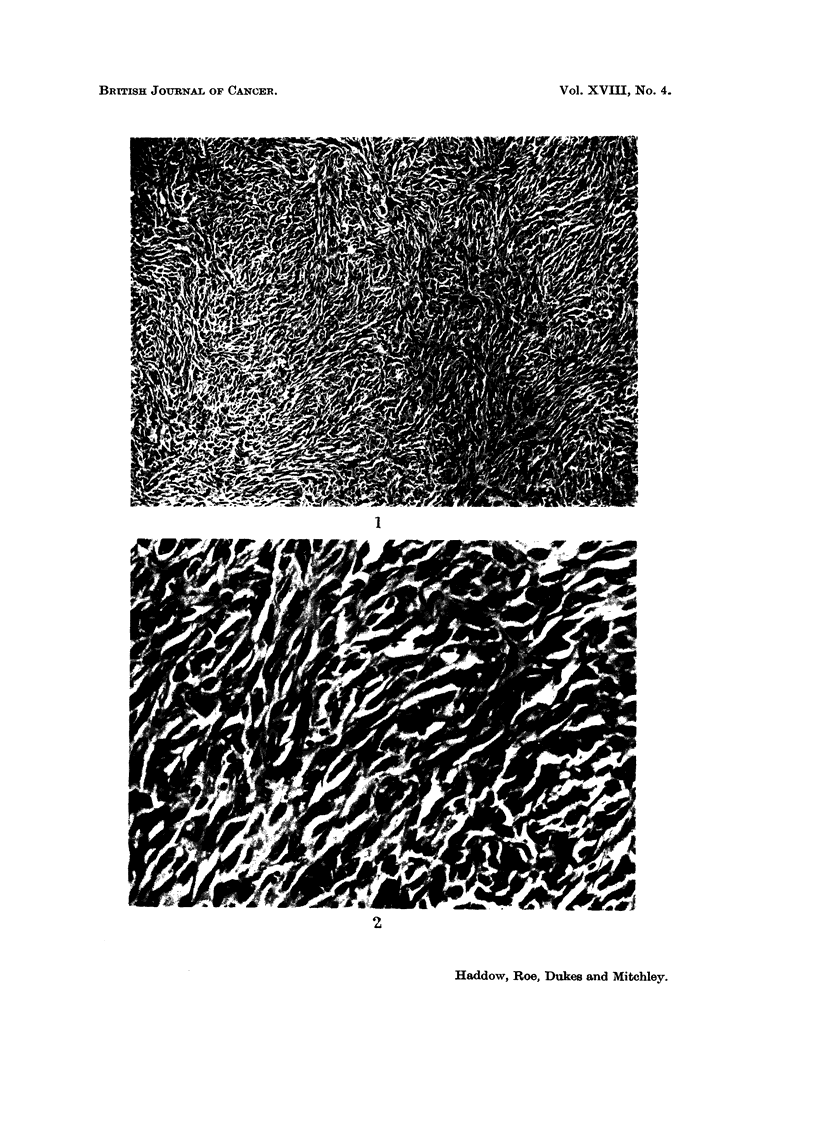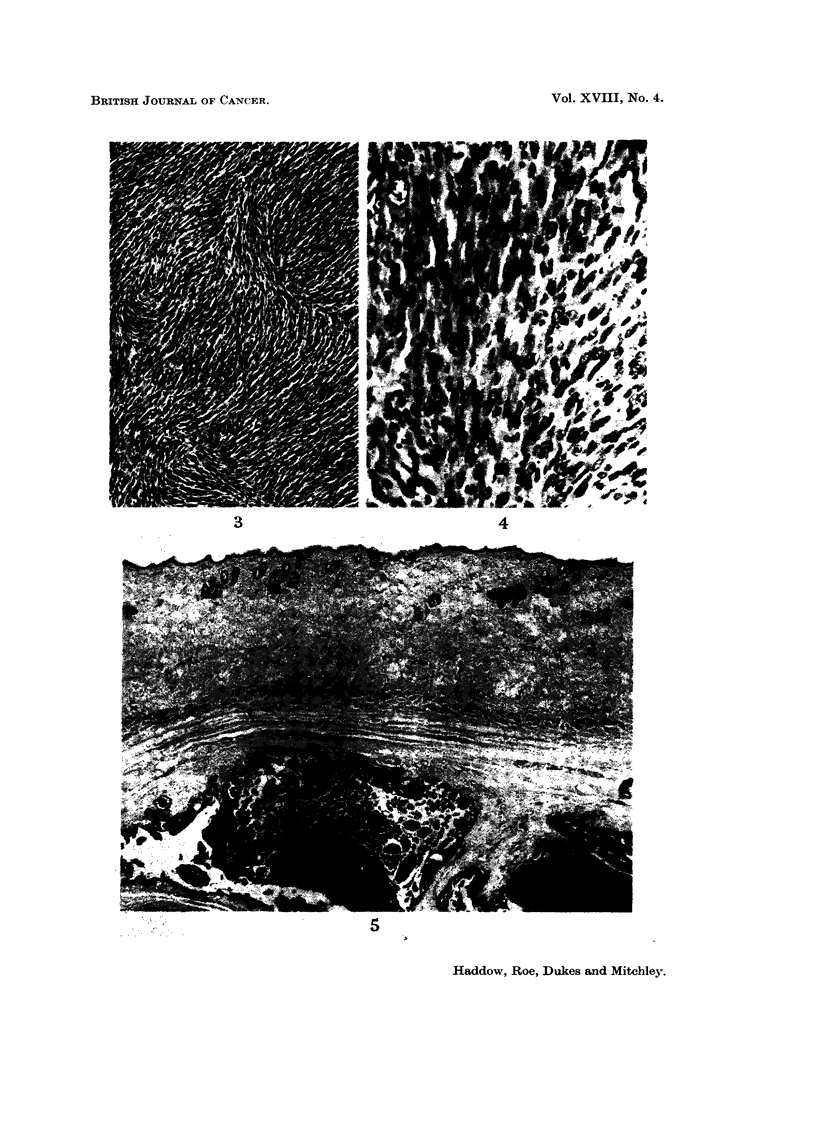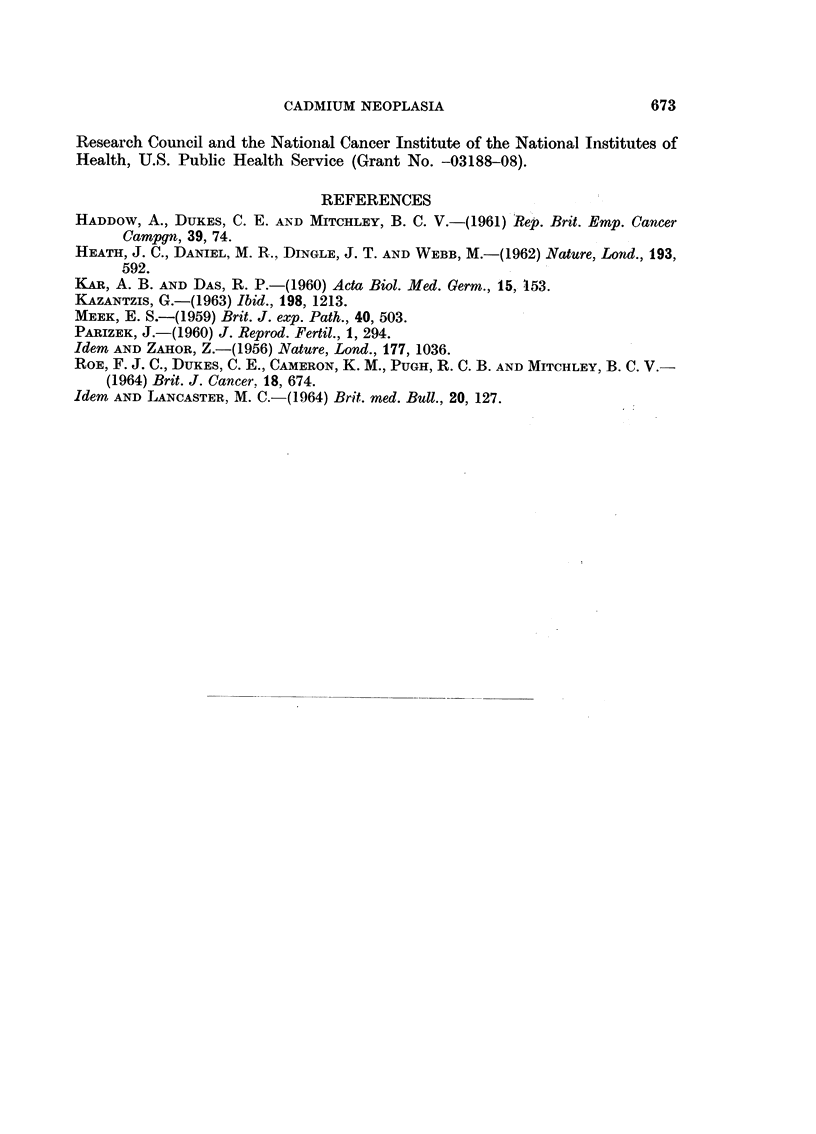# Cadmium Neoplasia: Sarcomata at the Site of Injection of Cadmium Sulphate in Rats and Mice

**DOI:** 10.1038/bjc.1964.76

**Published:** 1964-12

**Authors:** A. Haddow, F. J. C. Roe, C. E. Dukes, B. C. V. Mitchley

## Abstract

**Images:**


### CADMIUM NEOPLASIA : SARCOMATA AT THE SITE OF INJECTION OF CADMIUM SULPHATE IN RATS AND MICE

A. HADDOW, F. J. C. ROE, C. E. DUKES AND
B. C. V. MITCHLEY

*From the Chester Beatty Research Institute, Institute of Cancer Research : Royal Cancer Hospital, Fulham Road, London, S.W.3*

Received for publication August 7, 1964

In the course of a series of tests of iron-containing compounds and iron-complexes for carcinogenicity, it was found that a preparation of rat-ferritin induced not only a high incidence of sarcomata at the site of repeated subcutaneous injection, but also testicular atrophy, Leydig-cell hyperplasia and benign Leydig-cell tumours (Haddow, Dukes and Mitchley, 1961). The ferritin used in these experiments was prepared by the precipitation of rat liver protein by a cadmium salt, and contained cadmium as an essential part of its crystalline structure. Since cadmium is known to cause testicular atrophy (Parizek and Zahor, 1956 ; Meek, 1959 ; Kar and Das, 1960 ; Parizek, 1960) we decided to see whether the effects of rat ferritin could be induced by injections of a cadmium salt alone. Cadmium sulphate was the salt chosen for this purpose.

Recently, and at about the time the experiments to be described were being completed, two reports of the carcinogenicity of cadmium appeared. Heath *et al.* (1962) injected 20 hooded rats intramuscularly with cadmium powder suspended in fowl serum, 10 of the rats receiving 28 mg. each and 10 receiving 14 mg. each. Altogether 15 of the 20 rats developed malignant tumours : rhabdomyosarcomata and fibrosarcomata. Kazantzis (1963) injected 10 Wistar rats (Chester Beatty strain) subcutaneously at two sites with 25 mg. cadmium sulphide suspended in physiological saline. Sarcomata developed in 6 of the animals within 12 months. Neither of these authors described testicular changes in their experimental animals.

In this paper the induction of sarcomata at the site of subcutaneous injection of " rat-ferritin " or cadmium sulphate in rats is described and the failure to induce similar tumours in mice by injection of the same agents, reported. In an accompanying paper (Roe *et al.*, 1964) the testicular and pituitary changes following the injection of cadmium sulphate, or ferritin, are described and discussed.

### MATERIALS AND METHODS

*Rats :* Male albino rats of the Chester Beatty strain were used. In the first experiment courses of injection were begun when the rats were 3–4 weeks old (80–100 g.), and in the third experiment when they were 6–7 weeks old (170–200 g.). (Rats of this strain are exceptionally large.) The rats were housed in metal cages in groups of 10, fed cubed Diet 86 on 2 days of each week and white bread plus milk, cod liver oil, margarine, or marmite on the 5 other days. Water was provided *ad libitum*.

*Mice:* Random-bred Chester Beatty stock strain mice were used in Experiments II and IV. All mice were vaccinated on the tail with sheep lymph as a precaution against ectromelia. Injections were begun when the mice were 6 weeks old (25–35 g.). (Mice of this strain are exceptionally large.) Mice were housed in metal cages, 5 to a cage, fed Diet 41B and given water *ad libitum.*

*Preparation of ferritin.* The details of the preparation of ferritin are as follows :—

Two kilograms of rat's liver were collected and homogenised with water and heated to 80° C. to coagulate. The mass was then filtered through muslin and subsequently through a Whatman No. 1 filter paper. A clear brown solution was obtained.

To each 100 ml. of the above solution ammonium sulphate (A.R. grade) (30 g.) was added and the suspension allowed to stand at 4° C. in a refrigerator overnight. The resulting precipitate was collected by centrifugation. It was then dissolved in water and filtered, cadmium sulphate solution (4–5 g. per 100 ml.) added, and the whole kept at 4° C. in a refrigerator for 2 days. The resulting precipitate was collected by centrifugation and dried in a desiccator. The dried crude ferritin was re-crystallised by dissolving in ammonium sulphate solution (10 %) to which was added cadmium sulphate solution (4 %). After standing some time in the cold the ferritin was collected and rapidly washed with a saturated solution of potassium chloride.

The crystallisation of ferritin can be achieved by means of Zn, Cd, Ni or Co salts. Cadmium sulphate is the salt most commonly chosen for this. It seems necessary to have one of these elements present to form crystals. The cadmium may be considered to serve two functions ; the first, to co-ordinate the molecules of ferritin into a definite lattice pattern ; the second to decrease the solubility of ferritin, thus favouring crystallisation. The cadmium content of the re-crystallised ferritin was reduced by washing with a saturated potassium chloride solution, but some cadmium certainly remained in the final ferritin product used.

*Chemicals.* Crystalline cadmium sulphate with the formula $Cd\,SO_4.4H_2O$ was used, after a check had been made on its content of cadmium and water of crystallisation.

*Observation of animals.* Animals were examined regularly each week for the presence of tumours at the site of injection. They were killed when they became sick or developed rapidly growing injection-site tumours.

*Post-mortem examination.* All animals, whether killed because sick, or found dead, were subjected to careful post mortem examination. Organs showing pathological appearances were taken for histological examination.

### *Experiment I : Induction of Sarcomata at the Site of Subcutaneous Injection of Cadmium-precipitated Rat-Ferritin (in Rats)*

Twenty male rats, 3 weeks old, were injected subcutaneously in the right flank with ferritin prepared as above : the initial dose was 20 mg. but this caused severe ulceration at the injection site. A second dose of 20 mg. was given after an interval of 46 days and since this also caused a severe local reaction it was followed by eight doses of 2 mg. at weekly intervals, all given subcutaneously at the same site.

After 15 months one rat developed a palpable tumour at the site of injection

This proved to be a spindle cell sarcoma (Fig. 1 and 2). The only other lesion observed was atrophy of the seminiferous tubules in both testes. Fifteen of the remaining 19 rats were killed at intervals during the next 12 months, 6 of them with tumours at the site of injection. Material was taken for section from local tumours, both testes and other tissues in which abnormalities were noticed post mortem. Four rats in the series were " found dead " and too decomposed for histological investigations. However, as no visible or palpable tumour was present when these animals were examined during the 7 days preceding death, it may be presumed that they did not develop injection-site tumours.

Table I shows the incidence and time of appearance of tumours at the injection site. All seven injection-site tumours proved to be sarcomata. The testicular lesions are considered in detail in the accompanying paper (Roe *et al.*, 1964). No neoplasms of other organs were observed in this series of rats.

*Experiment II : Failure of Rat-Ferritin when Injected Subcutaneously into Mice, to induce Local Sarcomata*

Ten male stock mice, aged about 6 weeks, were given 3 subcutaneous injections into the right flank of 5 mg. rat ferritin at weekly intervals. Thereafter, because of inflammation and ulceration at the injection site, the animals were left for 6 weeks without treatment and the amount given in subsequent injections was reduced to 0·5 mg. A further 12 injections into the same flank of the reduced amount were given at weekly intervals. Eight of the mice died before the 10th month, one survived 13 months, and one almost 20 months. None developed tumours at the site of injection (Table I). One mouse developed generalised malignant lymphoma after 6 months. This condition occurs not infrequently in untreated mice of the strain used, and the occurrence of one case cannot be attributed to treatment in the present experiment. No other neoplasms were seen in any animal. At the time this experiment was undertaken there appeared to be no special reason for examining the testes at post mortem. Nevertheless, if gross changes in them had been present in any of the 10 male mice it is likely that they would have been observed. In fact none were noted.

*Experiment III : Induction of Sarcomata at the Site of Subcutaneous Injection of Cadmium Sulphate in Rats*

Twenty male rats, 6–7 weeks old, were given injections of 0·5 mg. cadmium sulphate in 1·0 ml. sterile distilled water subcutaneously into the right flank once weekly for 10 weeks. The total dose of metallic cadmium amounted to 2·0 mg. A group of 16 control male rats were kept under observation without treatment.

After 10½ months a tumour, which subsequently proved to be a spindle cell sarcoma, arose at the injection site of one of the test group. By the 20th month 14 out of the 20 rats in the group had developed similar tumours (Fig. 3 and 4). Apart from tumour formation extensive calcification was sometimes seen at the injection site. Fig. 5 illustrates this finding in a rat which failed to develop a tumour.

Testicular atrophy, Leydig-cell hyperplasia and neoplasia, and pituitary changes were seen in most of the cadmium-treated rats (see accompanying paper for details—Roe *et al.*, 1964). No significant changes were observed in any other organ.

Amongst the 16 untreated control rats the only neoplasm observed was a parenchymal-cell hepatoma. This was in a 12 months old rat. The survival of the control and treated rats was similar (Table I).

### *Experiment IV : Failure to Induce Sarcomata at the Site of Subcutaneous Injection of Cadmium Sulphate in Mice*

Twenty male stock mice, 6–7 weeks old, were given eleven once-weekly injections of 0·05 mg. cadmium sulphate in 0·2 ml. sterile distilled water subcutaneously into the right flank. The total dose of metallic cadmium amounted to 0·22 mg. A similar group of mice was kept under observation without treatment.

No injection-site tumours were observed in the cadmium-treated group but testicular atrophy, often with Leydig-cell hyperplasia, was present in almost all these animals (Roe *et al.*, 1964).

Generalised malignant lymphoma was observed in 4 of the cadmium-treated mice (in animals dying after 8, 13, 16 and 19 months, respectively), and a localised reticulosarcoma was seen in an animal killed during the 15th month. In the untreated controls generalised malignant lymphoma was seen in 3 animals (9, 17, and 19 months, respectively, from the beginning of the observation period, i.e. when the mice were 7 weeks old). In addition, a mouse dying during the 8th month had a parenchymal-cell tumour of the liver, one dying during the 9th month had a disseminated undifferentiated intra-abdominal adenocarcinoma of uncertain origin, and one dying during the 13th month had two small adenomatous tumours of the lung.

### DISCUSSION

A yield of 7 injection-site sarcomata in 20 rats injected with rat-ferritin after an average latent interval of 21·6 months is indicative of quite definite carcinogenicity of the injected material. On the other hand, the negative result obtained in 10 mice injected with a rather higher dose of rat-ferritin (on a body weight basis) is less meaningful, particularly because of their poor survival. After these experiments with rat-ferritin were complete it remained uncertain whether the injection-site tumours, and the testicular changes and neoplasms (Roe *et al.*, 1964) were due, as seemed likely, entirely to cadmium present in the ferritin or whether other constituents of the ferritin contributed to the effects seen. This problem persists since we have been unable so far to obtain a sample of ferritin which is free from cadmium. On the other hand, we have obtained further information on the carcinogenicity of cadmium in the absence of ferritin.

A total dose of 2·0 mg. Cd as $CdSO_4.4H_2O$ gave rise to 14 injection-site sarcomata in 20 rats. This incidence of tumours was double that obtained with approximately half the dose of cadmium (0·95 mg.) given as rat-ferritin and the average induction time was very much shorter : only 13·3 months as compared with 21·6 months. From this comparison it would seem that as far as the induction of local tumours is concerned, the other constituents of ferritin had little effect on carcinogenicity of cadmium.

In mice a total dose of cadmium (as cadmium sulphate) approximately equivalent, on a body weight basis, to that given to the rats (0·22 mg. as against 2·0 mg. for the rat) induced neither injection-site tumours nor testicular tumours (Roe *et al.*, 1964). The survival of the mice in this experiment was more satis-

TABLE I: *The Induction of Sarcomata at the Site of Subcutaneous Injection of Cadmium-Precipitated Rat-Ferritin, and of Cadmium Sulphate in Rats and Mice*

| Experiment | Species | No. of Animals | Treatment | Total Dose | Time of death (months) 0–10 | 10–12 | 12–14 | 14–16 | 16–18 | 18–20 | 20–22 | 22–24 | 24–26 | 26 + | Total yield of sarcomata | Average induction time (months) |
|---|---|---|---|---|---|---|---|---|---|---|---|---|---|---|---|---|
| I | Rat | 20 ♂ | 20 mg. rat-ferritin when animals 3 weeks old and similar dose when 9 weeks old: then 2 mg. doses once weekly for 8 weeks from 10th week onwards. All treatment given by S.C. injection into the right flank. | 56 mg Ferritin (≡0·95. mg. Cd) | | | | | ■ ?<br>○ ○ | ■ ■<br>? ○ | ■ ■<br>○ ?<br>○ | ■ ○ | ? ○<br>○ ○ | ■ | 7/20 | 21·6 |
| II | Mouse | 10 ♂ | 3 doses of 5 mg. rat-ferritin at weekly intervals from 6th week of age, then no treatment for 6 weeks. Thereafter 12 weekly injections of 0·5 mg. All treatments given by S.C. injection into the right flank. | 21 mg Ferritin (≡0·36 mg. Cd) | ○ ○<br>○ ○<br>○ ○<br>○ ○ | | ○ | | | ○ | | | | | 0/10 | |
| III | Rat | 20 ♂ | 10 once weekly injections of 0·5 mg. Cadmium Sulphate (Cd $SO_4 4H_2O$) in 1·0 ml. sterile distilled water subcutaneously into the right flank. | 5 mg Cadmium Sulphate (≡2·0 mg. Cd) | | ■ ■<br>■ ■<br>■ ■<br>○ | ■ ■<br>■ | ■ ■<br>○ | ○ ○<br>○ | ■ ■<br>■ ○ | | | | | 14/20 | 13·3 |
| | Rat | 16 ♂ (control) | None | None | ○ | ○ ○<br>○ | ○ | ○ ○<br>○ ○ | ○ ○<br>○ ○<br>○ ○ | | ○ | | | | 0/16 | |
| IV | Mouse | 20 ♂ | 11 once-weekly injections of 0·05 mg. Cadmium Sulphate (Cd $SO_4 4H_2O$) in 0·2 ml. sterile distilled water subcutaneously into the right flank. | 0·55 mg. Cadmium Sulphate (≡0·22 mg. Cd) | ○ ○ | ○ ○<br>○ ○<br>○ ○ | ○ ○<br>○ | ○ ○<br>○ | ○ ○<br>○ | ○ ○ | ○ | | | | 0/20 | |
| | Mouse | 20 ♂ (control) | None | None | ○ ?<br>○ ○<br>○ ○<br>○ | ○ ○<br>○ | ○ ○ | ? | ○ ○<br>○ ○ | ? ○<br>○ | | | | | 0/20 | |

■ = Died with histologically-proven sarcoma at injection site.

○ = Died without tumour at injection site.

? = No tumour seen when animal examined during the week preceding death; no histological sections of injection site available.

factory than in Experiment II, and the negativity of the result can be accepted with greater confidence.

In the experiments described, there was no indication that cadmium or cadmium-precipitated ferritin led to the induction of tumours other than at the site of injection and in the testis.

It is clear from the findings reported in this paper, and from those of others (see introduction), that cadmium must be included in the list of metals known to be capable of inducing cancer. This list now includes arsenic, beryllium, chromium, cobalt, iron (as certain iron-carbohydrate complexes), lead, nickel and zinc (see Roe and Lancaster, 1964, for review).

There appears at this stage to be a qualitative difference between the response of rats and mice to cadmium sulphate in so far as no neoplasm has yet been induced in the latter species, either at the injection site or in the testis. It is possible, however, that further experiments will prove this difference to be only a quantitative one.

### SUMMARY

Sarcomata arose at the site of repeated subcutaneous injections of cadmium sulphate or of cadmium-precipitated rat-ferritin in rats, but not in mice. The incidence of tumours was high (14 out of 20 rats) in response to cadmium sulphate, and the presence of ferritin seemed to have little effect on tumour induction.

The testicular changes, namely, atrophy of seminiferous tubules, Leydig-cell hyperplasia and Leydig-cell neoplasia, and the pituitary changes which also occurred in response to cadmium treatment are described and discussed in an accompanying paper (Roe *et al.*, 1964).

There was no indication in these experiments that the administration of cadmium or ferritin increased the incidence of neoplasms other than at the injection site or in the testis.

We are grateful to Mr. J. L. Everett for preparing the ferritin used in Experiments I and II and for measuring the cadmium and water content of the sample of cadmium sulphate used in Experiments III and IV. Our thanks are also due to Mr. E. Woollard and Mr. K. Moreman and their staff for histological and photographic assistance, respectively.

This investigation has been supported by grants to the Chester Beatty Research Institute (Institute of Cancer Research : Royal Cancer Hospital) from the Medical Research Council, the British Empire Cancer Campaign for Research, the Tobacco

---

### EXPLANATION OF PLATES

FIG. 1.—Spindle cell sarcoma arising in a rat at the site of the subcutaneous injection of cadmium-precipitated rat-ferritin. The tumour appeared after 15 months. H. & E. ×90.

FIG. 2.—Higher magnification of Fig. 1. H. & E. ×350.

FIG. 3.—Spindle cell sarcoma arising in a rat at the site of subcutaneous injection of cadmium sulphate. The tumour was noticed after 15 months and had metastasised to the lung. H. & E. ×90.

FIG. 4.—Higher magnification of Fig. 3. H. & E. ×350.

FIG. 5.—Calcification at site of subcutaneous injection of cadmium sulphate in a rat. This animal died during the 9th month of the experiment. No neoplasm was found at the injection site. H. & E. ×25.

1

2

Haddow, Roe, Dukes and Mitchley.

3

4

5

Haddow, Roe, Dukes and Mitchley.

Research Council and the National Cancer Institute of the National Institutes of Health, U.S. Public Health Service (Grant No. -03188-08).

### REFERENCES

HADDOW, A., DUKES, C. E. AND MITCHLEY, B. C. V.—(1961) *Rep. Brit. Emp. Cancer Campgn*, **39**, 74.
HEATH, J. C., DANIEL, M. R., DINGLE, J. T. AND WEBB, M.—(1962) *Nature, Lond.*, **193**, 592.
KAR, A. B. AND DAS, R. P.—(1960) *Acta Biol. Med. Germ.*, **15**, 153.
KAZANTZIS, G.—(1963) *Ibid.*, **198**, 1213.
MEEK, E. S.—(1959) *Brit. J. exp. Path.*, **40**, 503.
PARIZEK, J.—(1960) *J. Reprod. Fertil.*, **1**, 294.
*Idem* AND ZAHOR, Z.—(1956) *Nature, Lond.*, **177**, 1036.
ROE, F. J. C., DUKES, C. E., CAMERON, K. M., PUGH, R. C. B. AND MITCHLEY, B. C. V.—(1964) *Brit. J. Cancer*, **18**, 674.
*Idem* AND LANCASTER, M. C.—(1964) *Brit. med. Bull.*, **20**, 127.